# Effects of different vegetable oils on rumen fermentation and conjugated linoleic acid concentration *in vitro*

**DOI:** 10.14202/vetworld.2017.11-16

**Published:** 2017-01-08

**Authors:** Amitava Roy, Guru Prasad Mandal, Amlan Kumar Patra

**Affiliations:** Department of Animal Nutrition, West Bengal University of Animal and Fishery Sciences, Belgachia, Kolkata - 700 037, West Bengal, India

**Keywords:** conjugated linoleic acid, goat, rumen fluid, vaccenic acid, vegetable oil

## Abstract

**Aim:**

The objective of this study was to investigate the effect of different vegetable oils on rumen fermentation and concentrations of beneficial *cis*-9 *trans*-11 C18:2 conjugated linoleic acid (CLA) and *trans*-11 C18:1 fatty acid (FA) in the rumen fluid in an *in vitro* condition.

**Materials and Methods:**

Six vegetable oils including sunflower, soybean, sesame, rice bran, groundnut, and mustard oils were used at three dose levels (0%, 3% and 4% of substrate dry matter [DM] basis) in three replicates for each treatment in a completely randomized design using 6 × 3 factorial arrangement. Rumen fluid for microbial culture was collected from four goats fed on a diet of concentrate mixture and berseem hay at a ratio of 60:40 on DM basis. The *in vitro* fermentation was performed in 100 ml conical flakes containing 50 ml of culture media and 0.5 g of substrates containing 0%, 3% and 4% vegetable oils.

**Results:**

Oils supplementation did not affect (p>0.05) *in vitro* DM digestibility, and concentrations of total volatile FAs and ammonia-N. Sunflower oil and soybean oil decreased (p<0.05) protozoal numbers with increasing levels of oils. Other oils had less pronounced effect (p>0.05) on protozoal numbers. Both *trans*-11 C18:1 FA and *cis*-9, *trans*-11 CLA concentrations were increased (p<0.05) by sunflower and soybean oil supplementation at 4% level with the highest concentration observed for sunflower oil. The addition of other oils did not significantly (p>0.05) increase the *trans*-11 C18:1 FA and *cis*-9, *trans*-11 CLA concentrations as compared to the control. The concentrations of stearic, oleic, linoleic, and linolenic acids were not altered (p>0.05) due to the addition of any vegetable oils.

**Conclusion:**

Supplementation of sunflower and soybean oils enhanced beneficial *trans*-11 C18:1 FA and *cis*-9, *trans*-11 CLA concentrations in rumen fluid, while sesame, rice bran, groundnut, and mustard oils were ineffective in this study.

## Introduction

Enrichment of the nutraceutical quality of meat and milk of ruminant origins has been of growing interests among the researchers using dietary approaches due to increasing demands of healthy foods by the consumers [1-4]. The healthy fatty acids (FA), especially conjugated linoleic acids (CLAs) and n-3 polyunsaturated FA (eicosapentaenoic acid and docosahexaenoic acid) in foods for human consumption have shown several potential health benefits in several studies [1,5]. Another FA, *trans*-11 C18:1 (also called vaccenic acid; VA) is also associated with decreased risks of cardiovascular disease [6,7]. Milk and meat from ruminants are the main natural sources of CLAs [8]. However, usual dietary intakes of meat and milk are not adequate in fulfilling the requirement of *cis*-9, *trans*-11 CLA to achieve expected health benefits [1]. Therefore, several studies over the last two decades have been conducted for enhancing the *cis*-9, *trans*-11 CLA content in milk and meat of ruminants by supplementing linoleic and linolenic acid rich oils and oil seeds, which increase the availability of precursors of CLA synthesis [9,10], and modulating rumen microbiota and metabolism responsible for biohydrogenation of unsaturated C18 FA to stearic acid [2,3].

The *cis*-9, *trans*-11 CLA in meat and milk is partly absorbed from the gut after partial biohydrogenation of linoleic acid in the rumen [9]. The major part of this CLA is synthesized endogenously by the enzyme delta-9-desaturase in the animal tissues from VA, which is also a biohydrogenation intermediate of oleic, linoleic, and linolenic acids [11,12]. Different oils differ in their ability to increase the concentrations of these beneficial FA in rumen fluid and subsequently in meat and milk.

Therefore, this study was conducted to investigate the effects of different types of vegetable oils on rumen fermentation and concentration of CLAs and VA in rumen fluid *in vitro*.

## Materials and Methods

### Ethical approval

The experiment was approved by the Institutional Animal Ethics Committee for Animal Care and Management, West Bengal University of Animal and Fishery Sciences, Kolkata, West Bengal, India.

### Experimental design

Six different vegetable oils including sunflower, soybean, sesame, rice bran, groundnut, and mustard oils were procured from local grocery stores. These vegetable oils were used at three dose levels (0%, 3%, and 4% of substrate on dry matter [DM] basis) in three replicates for each treatment in a completely randomized design with a 6 × 3 factorial arrangement. Low concentrations of these oils as precursors of CLA in biohydrogenation process may not increase the CLA concentration in rumen fluid, while high concentration could inhibit overall rumen fermentation process [13]. We hypothesized that oils at 3-4% levels may enhance CLA concentration without affecting rumen fermentation.

### Rumen incubation

Rumen liquor was collected by a stomach tube from four goats fed on a diet of concentrate mixture and berseem hay at a 60:40 ratio on DM basis. The rumen liquor was collected during morning before feeding and watering, transported in insulated flasks under anaerobic conditions to the laboratory, pooled in equal proportions and used as a source of inoculums. The fermentation process was conducted in 100 ml conical flaskes containing 50 ml of culture media (1:4 ratio of rumen fluid and phosphate-bicarbonate buffer [14], purged with CO_2_), 0.5 g of substrates supplemented with 0%, 3%, 4% of each vegetable oil. Concentrate mixture (crude protein - 17.4%; neutral detergent fiber - 38.1%; ether extract - 1.12% on DM basis) and berseem hay (crude protein - 13.1%; neutral detergent fiber - 67.2%; ether extract - 1.21% on DM basis) at 60:40 ratio on DM basis were used as substrates. After flushing CO_2_ in the flasks for 5 min, a cork fitted with Bunsen gas release valve was tightly placed over the flasks and were incubated at 39°C for 24 h in a shaking incubator (105 rpm). After termination of incubation, the pH of the incubated media was determined by a digital pH meter, and then the content of the flasks was mixed properly and 1 ml of digestate was collected for protozoal counts. The remaining content was filtered through Gouch crucible (Grade I) and residual DM was analyzed to determine *in vitro* DM digestibility (IVDMD) as done earlier [15]. 15 ml of the filtrate was collected and stored at −20°C for further analysis.

### Laboratory analyses

For protozoa count, 1 ml sample was mixed with 1 ml methyl green formal saline solution. The stained sample was kept overnight, and protozoal numbers were counted microscopically using Neubauer counting chamber following the procedure of Kamra *et al*. [16]. Total volatile FA (VFA) was quantified as per the procedure of Barnett and Reid [17]. Ammonia-N was estimated by the micro-Kjeldahl method [18].

FA concentrations in feeds, vegetable oils, and rumen fluid were measured for control and 4% oil supplemented samples following the method of O’Fallon *et al*. [19] with slight modification, which has also been described previously [3]. 20 µl sample was placed into a 16 mm × 125 mm screw-cap Pyrex culture tube to which 0.5 ml of the C13:0 internal standard (0.5 mg of C13:0/mL of methanol), 0.35 ml of 10 N KOH in water, and 2.65 ml of MeOH were added. The tube was incubated at 55°C for 1.5 h with vigorous handshaking for 5 s every 20 min to properly permeate, dissolve and hydrolyze the FA in the samples. After cooling below room temperature in a cold tap water bath, 0.29 ml of 24 N H_2_SO_4_ in water was added. The tube was mixed by inversion and with precipitated K_2_SO_4_ present was incubated again at 55°C for 1.5 h with hand-shaking for 5 s every 20 min. After FA methyl ester (FAME) synthesis, the tube was cooled in a cold tap water bath, 1.5 ml of hexane was added, and the tube was vortex-mixed for 5 min on a multi-tube vortex. The tube was centrifuged for 5 min in a tabletop centrifuge at 2500 rpm, and the hexane layer, containing the FAME, was placed into a vial. The vial was capped and placed at −20°C until analysis. Concentrations of FA in the samples were analyzed in a gas chromatography fitted with capillary column (100 m × 0.25 mm × 0.20 µm). Helium was used as a carrier gas. FAs were identified by comparing their retention time with the FAME standard.

### Statistical analysis

The data analysis was performed by SPSS, version 16 [20] software. Rumen fermentation and FA concentration data were analyzed in two-way ANOVA with oil type, dose levels and their interaction as the main effects in 6 × 3 and 6 × 2 factorial arrangements, respectively. No variable except protozoal counts was affected (p>0.05) by the interaction effect. Then, data were analyzed in one-way ANOVA among the dose levels and oil type. Tukey’s test was used to find out the differences among the dose levels.

## Results

Mustard oil contained the highest concentration of C18:3 FA, while C18:2 FA content was higher in sunflower oil, followed by soybean oil (Table-1). Rice bran oil was richest in C18:1 and C16:0 FA. Ruminal fermentation parameters are presented in Table-2. Oils supplementation at 3% and 4% level did not influence IVDMD, pH, and concentrations of total VFA and ammonia-N in rumen fluid. Protozoal counts were affected by oil × level interaction. Sunflower oil and soybean oil decreased (p<0.05) protozoal numbers with increasing levels of oils (Table-3). Other oils had less pronounced effect on protozoal numbers. The effects of different vegetable oils on FA concentrations in rumen fluid are presented in Table-4. Both VA (*trans*-11 C18:1) and *cis*-9, *trans*-11 CLA concentrations were improved (p<0.05) by sunflower and soybean oil supplementation at 4% level. The increment of these FA was greater for sunflower oil. Addition of other oils did not significantly increase the VA concentrations as compared to the control. The concentrations of stearic, oleic, linoleic, and linolenic acids were not (p>0.05) altered due to addition of any vegetable oils.

**Table-1:** FA composition of vegetable oils and substrate (g/100 g of total FAs).

FA	Vegetable oil						Concentrate	Berseem

	Rice bran	Soybean	Sesame	Mustard	Sunflower	Groundnut		
C14:0	0.39	0.09	0.03	0.14	0.33	0.08	-	-
C16:0	20.7	12.2	9.73	4.16	8.50	13.1	16.8	19.0
C16:1	0.23	0.08	0.24	0.23	0.33	0.14	0.52	0.30
C18:0	2.66	3.27	5.10	1.83	7.40	4.00	7.21	5.03
C18:1	40.0	29.4	37.2	13.6	25.7	43.2	29.3	3.22
C18:2	34.4	45.8	39.6	34.0	54.2	35.8	45.9	11.3
C18:3	0.56	3.78	1.57	9.28	0.32	0.74	2.03	30.9

FA=Fatty acid

**Table-2:** Effects of vegetable oil supplementation on rumen fermentation in the rumen fluid after 24 h of incubation.

Vegetable oil	Dose level			SEM	p value

	0%	3%	4%		
IVDMD (%)					
Mustard	46.4	45.8	45.8	0.65	0.729
Groundnut	45.9	45.2	45.0	0.26	0.351
Sunflower	45.0	44.8	43.6	0.36	0.207
Sesame	46.0	45.8	45.7	0.35	0.531
Soybean	45.2	44.3	43.2	0.58	0.185
Rice bran	45.9	44.8	46.5	0.22	0.296
SEM	0.23	0.61	0.89		
p value	0.857	0.424	0.479		
Total VFA (mmol/dl)					
Mustard	5.58	5.42	5.50	0.05	0.640
Groundnut	5.42	5.38	5.44	0.04	0.554
Sunflower	5.37	5.36	5.15	0.05	0.361
Sesame	5.33	5.55	5.49	0.09	0.846
Soybean	5.42	5.28	5.46	0.08	0.262
Rice bran	5.39	5.45	5.32	0.05	0.450
SEM	0.07	0.08	0.10		
p value	0.778	0.692	0.623		
pH					
Mustard	6.67	6.63	6.53	0.03	0.813
Groundnut	6.53	6.56	6.67	0.04	0.732
Sunflower	6.60	6.57	6.40	0.05	0.708
Sesame	6.53	6.53	6.50	0.02	0.892
Soybean	6.53	6.47	6.50	0.04	0.655
Rice bran	6.53	6.63	6.56	0.04	0.647
SEM	0.08	0.05	0.06		
p value	0.904	0.718	0.754		
Ammonia-N (mg/dl)					
Mustard	8.32	8.09	8.27	0.05	0.424
Groundnut	8.07	8.20	8.30	0.06	0.375
Sunflower	8.07	8.20	8.30	0.06	0.408
Sesame	8.31	8.39	8.16	0.06	0.666
Soybean	8.38	8.06	8.26	0.07	0.307
Rice bran	8.34	7.96	8.09	0.08	0.266
SEM	0.09	0.08	0.05		
p value	0.670	0.512	0.540		

IVDMD=*In vitro* dry matter digestibility, VFA=Volatile fatty acids, SEM=Standard error of mean

**Table-3:** Effects of vegetable oil supplementation on protozoal population in the rumen liquor after 24 h of incubation.

Vegetable oil	Dose level			SEM	p value		
	
	0%	3%	4%		Oil	Dose level	Oil×dose
Mustard	36.2	35.2^x^	33.5^x^	1.21	0.264	0.005	0.108
Groundnut	35.3	36.2^x^	34.7^x^	0.61			
Sunflower	36.1^a^	31.5^by^	27.2^cy^	1.51			
Sesame	36.3	34.9^x^	34.3^x^	1.03			
Soybean	36.2^a^	33.2^abxy^	30.9^bxy^	1.19			
Rice bran	36.5	33.1^xy^	32.2^x^	0.91			
SEM	0.25	1.15	1.40				
p value	0.844	0.042	0.027				

a,b,c Means with different superscript letters within a row differ significantly (p<0.05).

xy Means with different superscripts letters within a column differ significantly (p<0.05). SEM=Standard error of mean

**Table-4:** Effect of vegetable oil supplementation on FA profile (% of total FAs) in the rumen fluid after 24 h of incubation.

Vegetable oil	Dose level		SEM	p value

	0%	4%		
Stearic acid (C18:0)				
Mustard	25.3	26.7	0.50	0.464
Groundnut	25.4	24.7	0.41	0.322
Sunflower	26.4	23.1	0.80	0.160
Sesame	26.2	25.8	0.60	0.377
Soybean	25.9	23.4	0.70	0.133
Rice bran	25.9	28.2	1.22	0.157
SEM	0.28	1.16		
p value	0.816	0.408		
Oleic acid (*cis*-9 C18:1)				
Mustard	15.5	18.2	0.90	0.106
Groundnut	15.8	18.3	0.64	0.089
Sunflower	16.7	13.9	1.30	0.154
Sesame	16.7	18.3	0.44	0.219
Soybean	16.7	15.6	1.00	0.431
Rice bran	17.0	14.4	0.80	0.136
SEM	0.46	1.53		
p value	0.613	0.340		
VA (*trans*-11 C18:1)				
Mustard	6.57	6.95^z^	0.13	0.709
Groundnut	6.66	7.10^z^	0.13	0.311
Sunflower	6.61^a^	9.34^b, x^	0.54	0.034
Sesame	6.65	7.15^z^	0.20	0.220
Soybean	6.74^a^	8.10^by^	0.33	0.014
Rice bran	6.65	7.36^yz^	0.18	0.192
SEM	0.06	0.85		
p value	0.772	0.026		
Linoleic acid (*cis*-9, *cis*-12 C18:2)				
Mustard	8.49	7.69	0.29	0.506
Groundnut	8.45	7.86	0.34	0.457
Sunflower	8.45	9.85	0.54	0.366
Sesame	8.50	7.66	0.24	0.234
Soybean	8.40	9.29	0.34	0.207
Rice bran	8.97	8.20	0.12	0.580
SEM	0.22	0.90		
p value	0.790	0.403		
CLA (*cis*-9, *trans*-11 C18:2)				
Mustard	0.31	0.35^z^	0.01	0.154
Groundnut	0.31	0.34^z^	0.01	0.137
Sunflower	0.32^a^	0.44^bx^	0.02	0.026
Sesame	0.31	0.35^z^	0.01	0.330
Soybean	0.31^a^	0.41^by^	0.02	0.019
Rice bran	0.31	0.36^z^	0.02	0.204
SEM	0.02	0.01		
p value	0.885	0.039		

a,b Means with different superscript letters in a row differ significantly (p<0.05).

x,y,z Means with different superscript letters in a column within a fatty acid differ significantly (p<0.05). SEM=Standard error of mean, CLA=Conjugated linoleic acid, VA=Vaccenic acid, FA=Fatty acid

## Discussion

Oil supplementation sometimes exerts detrimental effects on digestibility and VFA production due to general inhibitory effect of oils on rumen microbiota [21]. In this study, rumen fermentation was not affected, which was likely due to low concentration of oils used. Usually, oils or fats at concentrations of 4% in the diet do not affect rumen fermentation and may improve production performance of ruminants [13,22]. Soybean oil at 6% of diet did not influence DM degradability and total VFA concentrations *in vitro* [23]. Soybean oil-fish oil and rapeseed-fish oil blends reduced IVDMD when concentrations of oil were >5% of the diet, but not at lower concentration [24]. Sunflower oil and soybean oil inhibited the growth of protozoa, which was also observed in other studies [21] and is influenced by the degree of unsaturation of FA with greater unsaturation causing higher inhibitory effects on protozoa. Despite inhibition of protozoa, which may lower ammonia concentration by sunflower oil and soybean oil, ammonia concentration was not changed. This was probably due to lower time of incubation and low dose of oils to influence ammonia concentration by low number of protozoa. Gómez-Cortés *et al*. [23] reported that supplementation of soybean oil at 6% of the diet tended to decrease ammonia concentration *in vitro*.

Vegetable oils rich in C18:2 *cis*-9, *cis*-12 (linoleic acid) and C18:3 *cis*-9, *cis*-12, *cis*-15 (linolenic acid) FA could potentially increase VA and CLA concentration in the rumen fluid by bacteria biohydrogenation [10]. Linoleic acid has been shown to be converted to C18:2 *cis*-9, *tran*-11 and C18:1 *tran*-11 while linolenic acid can be converted to *cis*-9, *trans*-11, *cis*-15 C18:3 conjugated triene, then to *trans*-11, *cis*-15 C18:2, and finally to an octadecenoic acid that is either *trans*-11, *trans*-15 C18:2, or *cis*-15 C18:1 via rumen biohydrogenation [25]. The supplementation of soybean oil and sunflower oil enhanced VA and *cis*-9, *trans*-11 CLA concentrations in rumen fluid to a great extent. El-Sherbiny *et al*. [24] also found that VA and *cis*-9, *trans*-11 C18:2 concentrations in rumen fluid were increased by supplementation of soybean and rapeseed oil at 5% of DM, but not at 3% of DM. In another study, concentration of *cis*-9, *trans*-11 CLA was not altered, but the concentration of VA was increased in the *in vitro* rumen fluid by supplementation of soybean-fish oil blend at 3% of diet [26]. From this study and other studies, it appears that supplementation of vegetable oils at 4% or greater levels would be needed to achieve a significant effect on VA and *cis*-9, *trans*-11 CLA concentrations in rumen fluid *in vitro*. *In vivo* studies in lambs, heifers and goats have reported the enhancement of the CLA content in muscle and adipose tissue with addition of vegetable oils or seeds (safflower oil added up to 6% of the diet DM [27]; sunflower and linseed oils each at about 2.82% of diet DM [28]; sunflower and soybean oils each at 4.5% of the diet DM [29]). In lactating goats, the concentration of CLA in milk increased with safflower and linseed oil supplementation at 5% of diet [30]. Rice bran oil added in the concentrate mixture up to 6% linearly increased *cis*-9, *trans*-11 CLA and total CLA in milk of dairy cows [31]. Dai *et al*. [32] reported that the inclusion of vegetable oils (rapeseed, peanut, and sunflower seed oils each added at 2% of diet DM) increased the concentration of *cis*-9, *trans*-11 CLA. Increased VA and CLA concentrations in rumen fluid are attributed to partial biohydrogenation of linoleic and linolenic acid by ruminal microorganisms in response to oil supplementation [25]. Despite increases in concentrations of *cis*-9, *trans*-11 CLA and *trans*-11 C18:1 FA, the concentrations of C18:0 FA were not changed. The reason is unknown but it may be due to short duration of incubation. Again, concentrations of *cis*-9, *trans*-11 CLA were not significantly increased by sesame oil containing 39.6% C18:2 FA though soybean oil containing 45.8% C18:2 FA enhanced *cis*-9, *trans*-11 CLA in the rumen fluid. This may be attributed to the marginally low concentration of C18:2 FA in sesame oil. In a study by Dai *et al*. [32] also, peanut oil supplementation containing 26.9% C18:2 FA of total FA of diet increased *cis*-9, *trans*-11 CLA in milk of cows compared with rapeseed oil containing 31.6% C18:2 FA of total FA in diet DM. With other *in vitro* studies, this *in vitro* study has many shortcomings such as short incubation time, absence of digesta flow, low density of contents in the media, and continuous buffering activity, which would not represent true *in vivo* conditions. Nonetheless, *in vitro* study is useful to find out preliminary findings, which are required to be confirmed in long-term animal studies.

## Conclusion

Supplementation of vegetable oils rich in linoleic acid and linolenic acid such as sunflower oil and soybean oil at a dose of 4% of the diet could greatly increase beneficial *cis*-9, *trans*-11 CLA and VA concentrations in rumen fluid. These healthy FA after absorption from the intestine may be enriched in milk and meat of ruminants, but this should be confirmed *in vivo* animal experimentations.

## Authors’ Contributions

GPM and AR carried out the experiment design. AR participated in practical work. AKP and GPM performed statistical analysis, data interpretation and writing of the manuscript. All authors read and approved the final manuscript.
